# Uncovering the protective mechanism of Taohong Siwu decoction against diabetic retinopathy via HIF-1 signaling pathway based on network analysis and experimental validation

**DOI:** 10.1186/s12906-020-03086-0

**Published:** 2020-10-06

**Authors:** Lei Wang, Shuyan Li, Leilei Wang, Kai Lin, Jialun Du, Wanhong Miao, Lei Zhang

**Affiliations:** 1 The Seventh People’s Hospital of Shanghai University of Traditional Chinese Medicine, 358 Datong Road, Pudong, Shanghai, 200137 China; 2 Shanghai University of Traditional Chinese Medicine, 1200 Cailun Road, Shanghai, 201203 China; 3 Shuguang Hospital, Shanghai University of Traditional Chinese Medicine, 528 Zhangheng Road, Shanghai, 201203 China

**Keywords:** Taohong Siwu decoction, Diabetic retinopathy, HIF-1 signaling pathway, Network pharmacology, Angiogenesis

## Abstract

**Background:**

Diabetic retinopathy (DR) is a common and serious microvascular complication of diabetes. Taohong Siwu decoction (THSWD), a famous traditional Chinese medicine (TCM) prescription, has been proved to have a good clinical effect on DR, whereas its molecular mechanism remains unclear. Our study aimed to uncover the core targets and signaling pathways of THSWD against DR.

**Methods:**

First, the active ingredients of THSWD were searched from Traditional Chinese Medicine Systems Pharmacology (TCMSP) Database. Second, the targets of active ingredients were identified from ChemMapper and PharmMapper databases. Third, DR associated targets were searched from DisGeNET, DrugBank and Therapeutic Target Database (TTD). Subsequently, the common targets of active ingredients and DR were found and analyzed in STRING database. DAVID database and ClueGo plug-in software were used to carry out the gene ontology (GO) and KEGG enrichment analysis. The core signaling pathway network of “herb-ingredient-target” was constructed by the Cytoscape software. Finally, the key genes of THSWD against DR were validated by quantitative real-time PCR (qRT-PCR).

**Results:**

A total of 2340 targets of 61 active ingredients in THSWD were obtained. Simultaneously, a total of 263 DR-associated targets were also obtained. Then, 67 common targets were found by overlapping them, and 23 core targets were identified from protein-protein interaction (PPI) network. Response to hypoxia was found as the top GO term of biological process, and HIF-1 signaling pathway was found as the top KEGG pathway. Among the key genes in HIF-1 pathway, the mRNA expression levels of *VEGFA*, *SERPINE1* and *NOS2* were significantly down-regulated by THSWD (*P* < 0.05), and *NOS3* and *HMOX1* were significantly up-regulated (*P* < 0.05).

**Conclusion:**

THSWD had a protective effect on DR via regulating HIF-1 signaling pathway and other important pathways. This study might provide a theoretical basis for the application of THSWD and the development of new drugs for the treatment of DR.

## Background

Diabetic retinopathy (DR) is a common and serious microvascular complication of diabetes, which is an important cause of permanent vision loss in middle-aged and elderly people. One-third of people with diabetes have DR. [1] Previous study reported that 2.6 million people were visually impaired (moderate to severe vision impairment or even blindness) resulting from DR in 2015 and it was estimated to rise to 3.2 million in 2020 [2]. Furthermore, DR not only reduces the quality of life, but also increases the risk of other diabetes complications and even mortality, which brings severe social and family burdens [3]. Currently, the therapeutic approaches of DR mainly include surgery, laser photocoagulation, hormone, and anti-vascular endothelial growth factor (VEGF) drug. However, the current treatment could bring some adverse reactions, such as high intraocular pressure, angiogenesis, retinal hemorrhage, and so on [4, 5]. Therefore, it is urgent to discover promising therapeutic targets and develop new therapeutic strategies for DR patients.

The pathogenesis of DR is complicated because multiple factors induce the disease. Currently, it is believed that DR is closely associated with glucose metabolism and microvascular status. In specific, glucose metabolic disorder will lead to microvascular alternation and microcirculation dysfunction, subsequently retina ischemia and hypoxia, and finally occurrence of retinopathy [6, 7]. Previous studies reported that hypoxia played an essential role in DR progression via promoting neovascularization and vascular dystrophies [8–10]. Some researchers found that the retinas of diabetic patients could quickly response to hyperglycemia and hypoxia, resulting in the imbalance between pro-angiogenesis and anti-angiogenesis [11]. Thus, the treatments which focus on hypoxia-related signaling pathways and targets may be the novel and promising therapeutic strategies for DR.

Taohong Siwu decoction (THSWD) is a famous traditional Chinese medicine (TCM) prescription, and was first recorded in a well-known medical book Yizong Jinjian compiled by Wu Qian in Qing Dynasty [12]. It consists of six herbs, including Persicae Semen (Taoren, TR), Carthami Flos (Honghua, HH), Rehmanniae Radix Praeparata (Shudihuang, SHH), Angelicae Sinensis Radix (Danggui, DG), Paeoniae Radix Alba (Baishao, BS), and Chuanxiong Rhizoma (Chuanxiong, CX) (Table 1). Several studies have reported that THSWD had a good clinical effect on the treatment of DR. [13] However, the pharmacological mechanism of THSWD on DR therapy remains unclear, which restricts the wide use of THSWD.

**Table 1:** The ingredients of THSWD

Chinese name	Pharmaceutical name	Botanical plant name	English name
Tao Ren	Persicae Semen	*Prums persica* (L.) Batsch	Peach Seed
Hong Hua	Carthami Flos	*Carthamus tinctorius* L.	Safflower
Shu Di Huang	Rehmanniae Radix Praepata	*Rehmannia glutinosa* Libosch.	Chinese Fox-Glove Root
Dang Gui	Angelicae Sinensis Radix	*Angelica sinensis* (Oliv.) Diels	Chinese Angelica
Bai Shao	Paeoniae Radix Alba	*Paeonia lactiflora* Pall.	White Peony Root
Chuan Xiong	Chuanxiong Rhizoma	*Ligusticum chuanxiong* Hort.	Szechwan Lovage Rhizome

**Fig. 1:**
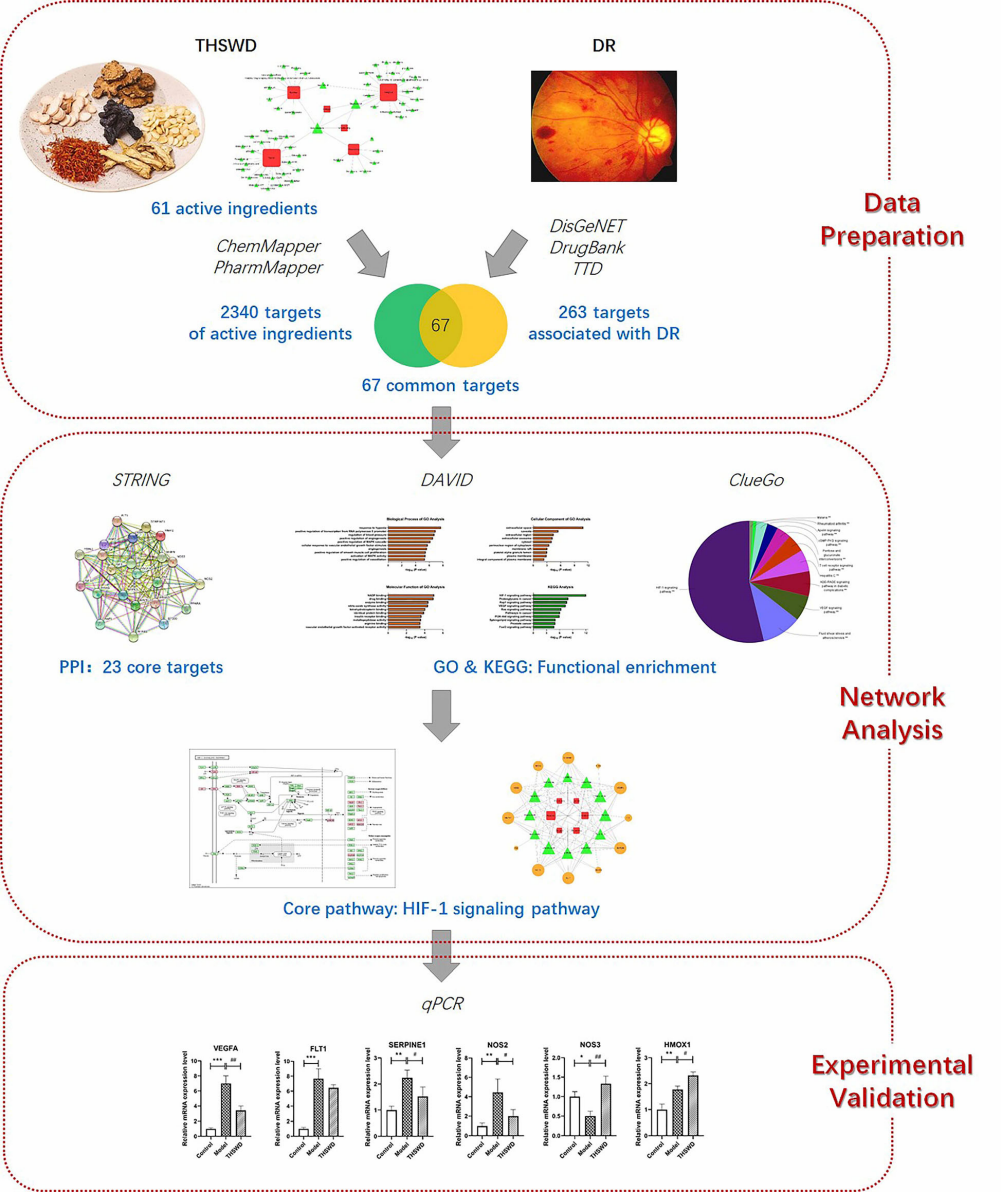
The flowchart for exploring the molecular mechanisms of THSWD against DR

In this study, network pharmacology and experimental validation were conducted to explore the mechanism of THSWD on DR therapy (Fig. 1). Firstly, the targets of THSWD against DR were searched and screened. Subsequently, network analysis was applied to explore the core signaling pathways and targets. Finally, in vitro experiments were performed to validate the molecular mechanism of THSWD on the treatment of DR, and hypoxia inducible factor-1 (HIF-1)-related pathways and targets were found involved in THSWD against DR. Our study maybe explains the therapeutic mechanism of THSWD at the molecular level and provides the theoretical basis for the wide use of THSWD on the treatment of DR.

## Methods

### Data preparation

#### Searching for active ingredients of THSWD

Ingredients of each herb in THSWD were searched from Traditional Chinese Medicine Systems Pharmacology (TCMSP) Database (http://lsp.nwu.edu.cn/tcmsp.php), a unique pharmacology platform that collects a great deal of information about herbal ingredients, targets and disease [14]. The active ingredients were filtered by meeting the criteria of oral bioavailability (OB ≥ 30%) and drug-likeness (DL ≥ 0.18) as previous study [15]. An interaction network of herb-ingredient was constructed and visualized by the Cytoscape software (version 3.2.1, Boston, MA, USA).

#### Identification of targets of active ingredients

ChemMapper (http://lilab.ecust.edu.cn/chemmapper/) and PharmMapper (http://lilab.ecust.edu.cn/pharmmapper/index.php) databases were used to predict the targets of each active ingredient. ChemMapper database is a versatile web server for exploring pharmacology and chemical structure association based on molecular 3D similarity method [16]. The screening criteria were 3D structure similarity above 0.85 and prediction score above 0. PharmMapper database is another web server for identifying potential drug targets according to the target pharmacophore approach [17]. The druggable pharmacophore models were set and the top 100 reserved matched targets were selected with default parameters.

#### Searching for DR-associated targets

DR-associated targets were searched with keywords “diabetic retinopathy”, “proliferative diabetic retinopathy”, and “nonproliferative diabetic retinopathy” in DisGeNET database (http://www.disgenet.org/web/DisGeNET/menu/home), DrugBank database (https://www.drugbank.ca/), and Therapeutic Target Database (TTD) (https://db.idrblab.org/ttd/) which contain collections of genes and variants associated to human diseases [18–20].

### Network analysis

#### Construction and analysis of protein-protein interaction (PPI)

The PPI of candidate targets of THSWD against DR were collected from STRING database (https://string-db.org/), which integrates the quality-controlled protein-protein association networks of a large number of organisms [21]. The criteria for PPI selection were those with the confidence score above 0.9 and the degree score above the average value.

#### Functional enrichment analysis

The candidate targets of THSWD against DR were imported into DAVID database (https://david.ncifcrf.gov/) for the Gene Ontology (GO) and the Kyoto Encyclopedia of Genes and Genomes (KEGG) analysis. DAVID database provides systematic and comprehensive bioinformatics annotations for large-scale gene or protein lists based on both biological data and analysis tools [22].

The candidate targets of THSWD against DR were further analyzed with a Cytoscape plug-in software, ClueGo. GlueGo was used to decipher functionally grouped gene ontology and pathway annotation networks [23]. *P* < 0.05 was regarded as the significant cutoff in this study.

#### Pathway network construction

Based on the data of herb-ingredient-target-pathway obtained by previous steps, the core signaling pathway network of THSWD against DR was constructed by the Cytoscape software. The critical active ingredients and candidate targets were filtered based on the criterion of degree score above the average value.

### Experimental validation

#### Liquid chromatography/mass spectrometry (LC/MS) analysis

A liquid chromatography-mass spectrometry system, AB Sciex Triple TOF® 4600, was used for the separation and identification of THSWD. The separation was performed on a Waters ACQUITY UPLC HSS T3 column (2.1 × 100 mm, 1.8 μm, Lot: 0200372571). A linear gradient elution of A (0.1% formic acid-H_2_O) and B (acetonitrile) was used with the gradient procedure as list in Table 2. DAD was on and the target wavelength was simultaneously set at 254 nm. MS parameters were set as list in Table 3. Approximately 1 ml of the sample was dissolved in 10 mL water. The solution was centrifuged at 12000 rpm for 5 min, and the supernatants of sample solutions were filtered through a 0.22 μm membrane filter prior to LC/MS analysis.

**Table 2:** Mobile phase gradient

Time (min)	Flow Rate (ml/min)	%B
0	0.3	0
8	0.3	0
10	0.3	3
28	0.3	10
35	0.3	15
43	0.3	20
48	0.3	25
55	0.3	40
62	0.3	60
68	0.3	95
73	0.3	95
73.1	0.3	0
76	0.3	0

**Table 3:** Mass parameters

MS parameters	parameter	MS/MS parameter	parameter
TOF mass range	100 ~ 1500	MS/MS mass range	100 ~ 1500
Ion Source Gas 1	50	Declustering Potential	100
Ion Source Gas 2	50	Collision Energy	±40
Curtain Gas	35	Collision Energy Spread	20
Ion Spray Voltage Floating (kV)	− 4500/5000	Ion Release Delay	30
Ion Source Temperature (°C)	500	Ion Release Width	15
Declustering Potential	100		
Collision Energy	10		

#### Cell culture and treatment

Human retinal microvascular endothelial cells (hRMECs) were purchased from Shanghai Bioleaf Biotech Co., Ltd. (Shanghai, China) and cultured in endothelial cell medium containing 5% fetal bovine serum, supplemented with 1% penicillin-streptomycin and 1% endothelial cell growth supplement (Gibco, Thermo Fisher Scientific, Inc., Waltham, MA, USA) at 37 °C with 5% CO_2_. hRMECs were treated with 30 mM glucose alone to simulate DR or in combination with 100 μg/ml THSWD for 24 h. Cells without any treatments were used as control.

#### Quantitative real-time PCR (qRT-PCR)

Total RNA was extracted using TRIzol Reagent (Invitrogen, Carlsbad, CA, USA). The quality of RNA was measured by Nanodrop 2000 (Thermo Scientific, Rockford, IL, USA), and equal amounts of RNA were reverse-transcribed into cDNA using First-Strand cDNA Synthesis kits (Invitrogen, Carlsbad, CA, USA). Gene primer pairs used in this study are list in Table 4. qRT-PCR was carried out using ABI 7500 System (Applied Biosystems, Foster City, CA, USA) under the following parameters: 95 °C for 30s, 95 °C for 5 s (40 cycles), 60 °C for 30s, and 72 °C for 15 s. Relative mRNA expression levels were calculated using *GAPDH* as the internal control and the 2^-△△CT^ method. Each sample was run three times.

**Table 4:** Gene primer pairs used for qRT-PCR

Gene	Forward	Reverse
*VEGFA*	5′-GAGCCTTGCCTTGCTGCTCTAC-3′	5′-CACCAGGGTCTCGATTGGATG-3′
*FLT1*	5′-GCATATGGTATCCCTCAACCTACAA-3′	5′-CATCCAGGATAAAGGACTCTTCATTAT-3′
*SERPINE1*	5′-CAGACCAAGAGCCTCTCCAC-3′	5′-GGTTCCATCACTTGGCCCAT-3′
*NOS2*	5′-CGGTGCTGTATTTCCTTACGAGGCGAAGAAGG-3′	5′-GGTGCTGCTTGTTAGGAGGTCAAGTAAAGGGC-3′
*NOS3*	5′-GACCCTCACCGCTACAACAT-3′	5′-CCGGGTATCCAGGTCCAT-3′
*HMOX1*	5′-CTGGGCTGGGGACAGTGG-3′	5′-GAAAAGAGAGCCAGGCAAGAT-3′
*GAPDH*	5′-CGCTCTCTGCTCCTCCTGTT-3’	5′-CCATGGTGTCTGAGCGATGT-3’

### Statistical analysis

Quantitative data were presented as mean ± standard deviation (SD). One-way ANOVA analysis, followed by Dunnett post hoc test, was used to determine significant differences between different groups using the SPSS software (version 21.0, Chicago, IL, USA). *P* < 0.05 was considered statistically significant.

## Results

### Candidate targets of THSWD against DR

#### Active ingredients of THSWD

**Fig. 2:**
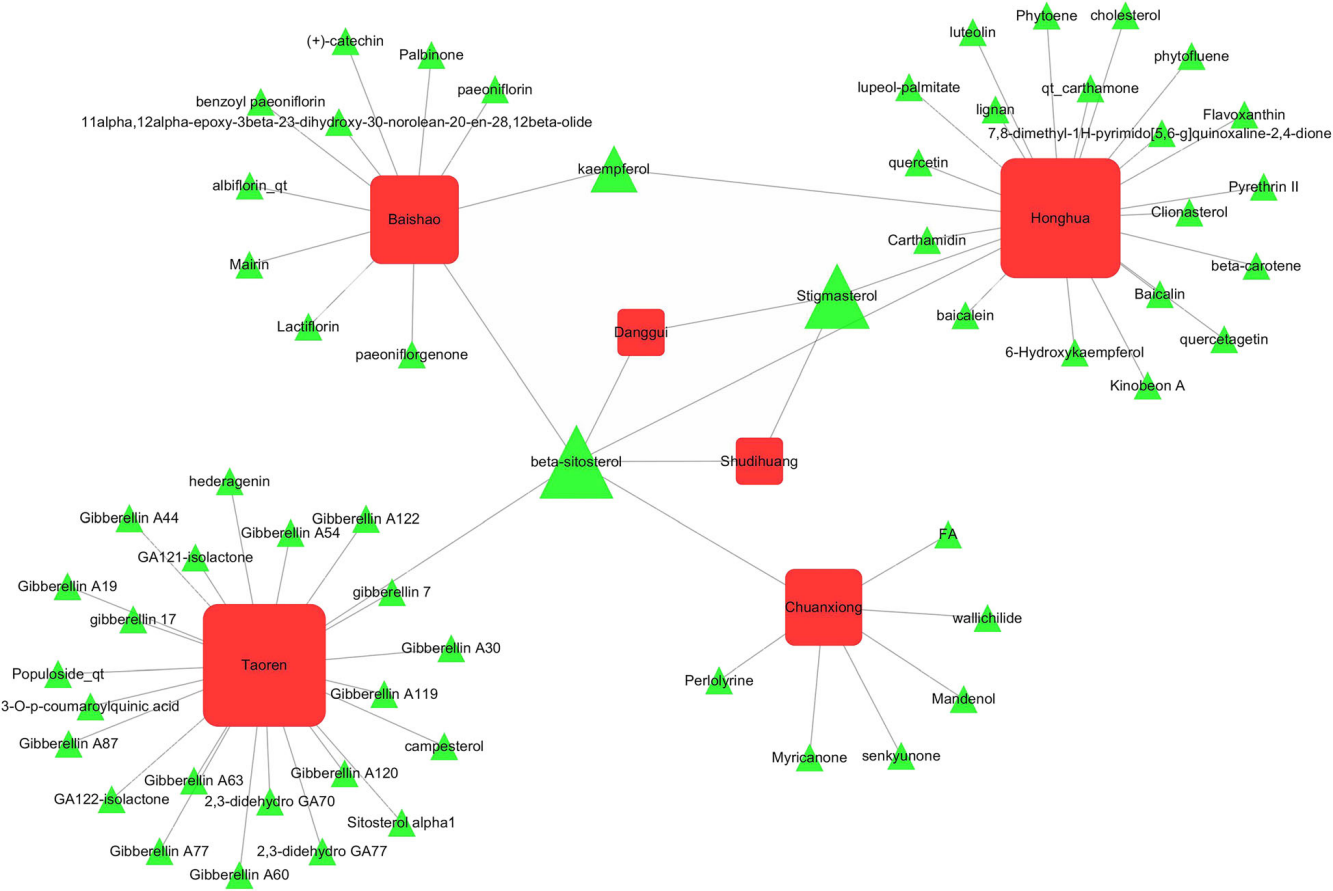
Sixty one active ingredients obtained in THSWD. The red square represents herbs in THSWD, and the green triangle represents active ingredients of those herbs

A total of 722 ingredients in THSWD were found from TCMSP database. According to the screening criteria of OB ≥ 30% and DL ≥ 0.18, we filtered out 23 active ingredients in TR, 22 active ingredients in HH, 2 active ingredients in SDH, 2 active ingredients in DG, 13 active ingredients in BS, and 7 active ingredients in CX. After overlapped ingredients were subtracted, a total of 61 active ingredients in THSWD were obtained. Detailed information about active ingredients of THSWD was provided in Fig. 2 and Supplementary Table 1.

#### Targets of active ingredients in THSWD

We found 1567 targets of TR, 1656 targets of HH, 468 targets of SDH, 741 targets of DG, 1567 targets of BS, and 1307 targets of CX. After overlapped targets were subtracted, a total of 2340 targets of THSWD were obtained, including 1186 targets from ChemMapper database and 1182 from PharmMapper database. Detailed information about targets of active ingredients in THSWD was provided in Supplementary Table 2.

#### DR-associated targets

After overlapped targets were subtracted, a total of 263 DR-associated targets were obtained, including 258 DR-associated targets from DisGeNET Database, 3 ones from DrugBank database, and 7 ones from TTD database. Detailed information about DR-associated targets was provided in Supplementary Table 3.

#### Targets of THSWD against DR

The targets of active ingredients in THSWD were overlapped with DR-associated targets. A total of 67 candidate targets of THSWD against DR were identified. Detailed information about targets of THSWD against DR was provided in Table 5.

**Table 5:** Targets of THSWD against DR

UniProt ID	Gene Symbol	Gene Name
P20248	CCNA2	cyclin A2
P12821	ACE	angiotensin I converting enzyme
P09488	GSTM1	glutathione S-transferase mu 1
P08473	MME	membrane metalloendopeptidase
P09038	FGF2	fibroblast growth factor 2
Q07869	PPARA	peroxisome proliferator activated receptor alpha
P15692	VEGFA	vascular endothelial growth factor A
P04406	GAPDH	glyceraldehyde-3-phosphate dehydrogenase
Q04760	GLO1	glyoxalase I
P37231	PPARG	peroxisome proliferator activated receptor gamma
P35354	PTGS2	prostaglandin-endoperoxide synthase 2
P01112	HRAS	HRas proto-oncogene, GTPase
P29475	NOS1	nitric oxide synthase 1
P08069	IGF1R	insulin like growth factor 1 receptor
P30556	AGTR1	angiotensin II receptor type 1
P05019	IGF1	insulin like growth factor 1
P27361	MAPK3	mitogen-activated protein kinase 3
P01375	TNF	tumor necrosis factor
P14780	MMP9	matrix metallopeptidase 9
P05091	ALDH2	aldehyde dehydrogenase 2 family (mitochondrial)
P05121	SERPINE1	serpin family E member 1
Q00796	SORD	sorbitol dehydrogenase
P22392	NME2	NME/NM23 nucleoside diphosphate kinase 2
P00403	MT-CO2	cytochrome c oxidase subunit II
Q16698	DECR1	2,4-dienoyl-CoA reductase 1, mitochondrial
P14550	AKR1A1	aldo-keto reductase family 1 member A1
P09601	HMOX1	heme oxygenase 1
P15121	AKR1B1	aldo-keto reductase family 1 member B
P29474	NOS3	nitric oxide synthase 3
Q16539	MAPK14	mitogen-activated protein kinase 14
O60218	AKR1B10	aldo-keto reductase family 1 member B10
P18031	PTPN1	protein tyrosine phosphatase, non-receptor type 1
P55017	SLC12A3	solute carrier family 12 member 3
P11226	MBL2	mannose binding lectin 2
P21912	SDHB	succinate dehydrogenase complex iron sulfur subunit B
P07339	CTSD	cathepsin D
P17405	SMPD1	sphingomyelin phosphodiesterase 1
P14210	HGF	hepatocyte growth factor
P16581	SELE	selectin E
P16109	SELP	selectin P
P09238	MMP10	matrix metallopeptidase 10
P08254	MMP3	matrix metallopeptidase 3
P12724	RNASE3	ribonuclease A family member 3
P08253	MMP2	matrix metallopeptidase 2
P35228	NOS2	nitric oxide synthase 2
P51606	RENBP	renin binding protein
O60909	B4GALT2	beta-1,4-galactosyltransferase 2
P21554	CNR1	cannabinoid receptor 1
P13945	ADRB3	adrenoceptor beta 3
P08100	RHO	rhodopsin
P01308	INS	insulin
O00206	TLR4	toll like receptor 4
Q08209	PPP3CA	protein phosphatase 3 catalytic subunit alpha
P30711	GSTT1	glutathione S-transferase theta 1
P19440	GGT1	gamma-glutamyltransferase 1
P35968	KDR	kinase insert domain receptor
P35916	FLT4	fms related tyrosine kinase 4
P17948	FLT1	fms related tyrosine kinase 1
P04049	RAF1	Raf-1 proto-oncogene, serine/threonine kinase
P42898	MTHFR	methylenetetrahydrofolate reductase
P19838	NFKB1	nuclear factor kappa B subunit 1
P20132	SDS	serine dehydratase
P10745	RBP3	retinol binding protein 3
P52757	CHN2	chimerin 2
P17707	AMD1	adenosylmethionine decarboxylase 1
P04179	SOD2	superoxide dismutase 2, mitochondrial
Q09472	EP300	E1A binding protein p300

### Key targets and signaling pathway of THSWD against DR

#### Core targets in protein-protein interaction (PPI)

**Fig. 3:**
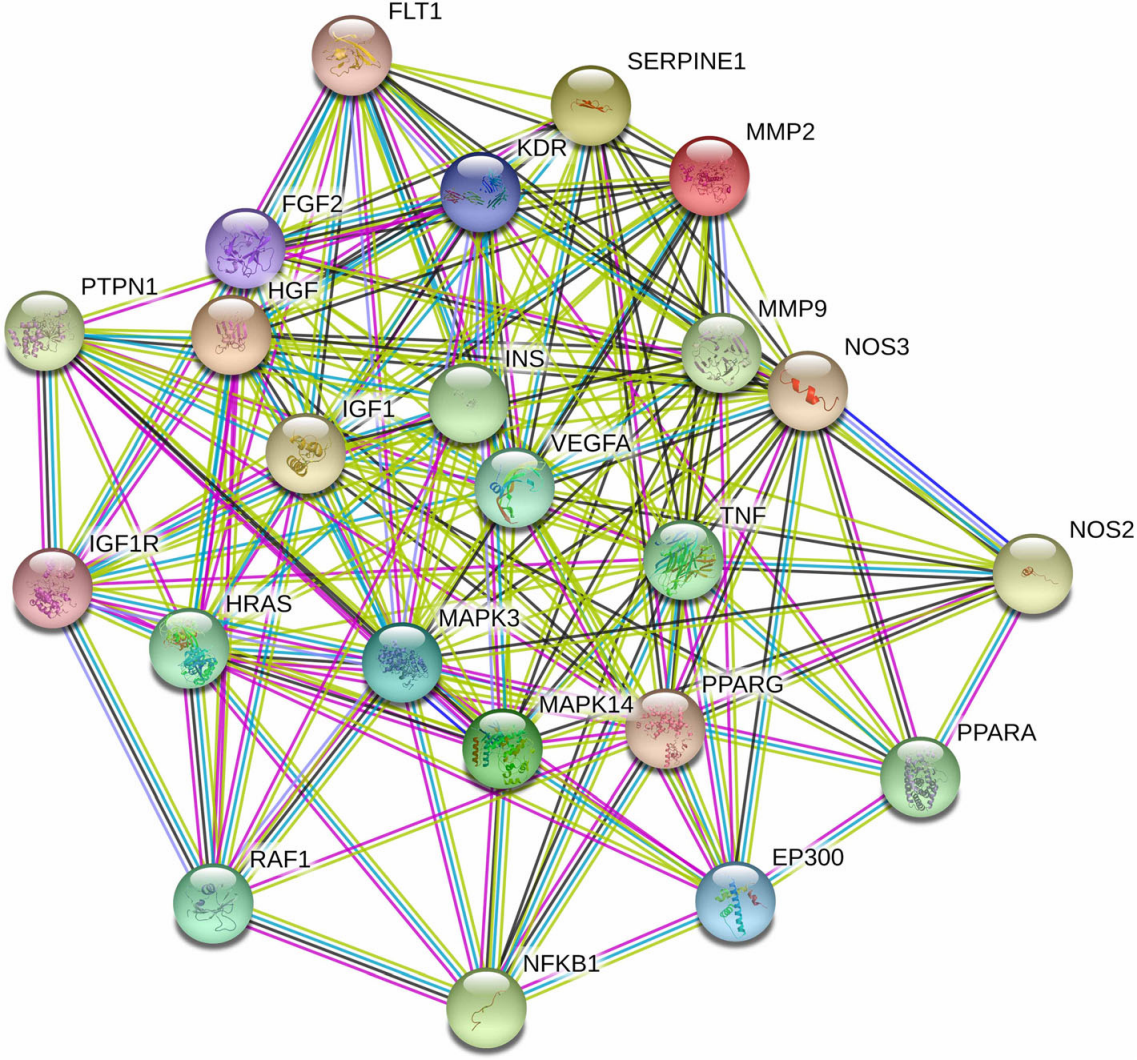
PPI network of 23 core targets of THSWD against DR. In the PPI diagram, each solid circle represents a target, and the middle of the circle shows the structure of the protein

The 67 candidate targets mentioned above were imported into STRING database to build the PPI network. According to the screening criteria of the confidence score above 0.9 and the degree score above the average value, 23 core targets were filtered out, including VEGFA, TNF, IGF1, INS, MAPK3, NFKB1, EP300, HRAS, FGF2, HGF, IGF1R, KDR, MAPK14, MMP9, MMP2, PPARA, RAF1, FLT1, NOS2, NOS3, PPARG, PTPN1 and SERPINE1 (Fig. 3). These 23 core targets may be used as valuable targets in the treatment of THSWD against DR and deserve further study.

#### Functional enrichment of GO terms and KEGG pathways

**Fig. 4:**
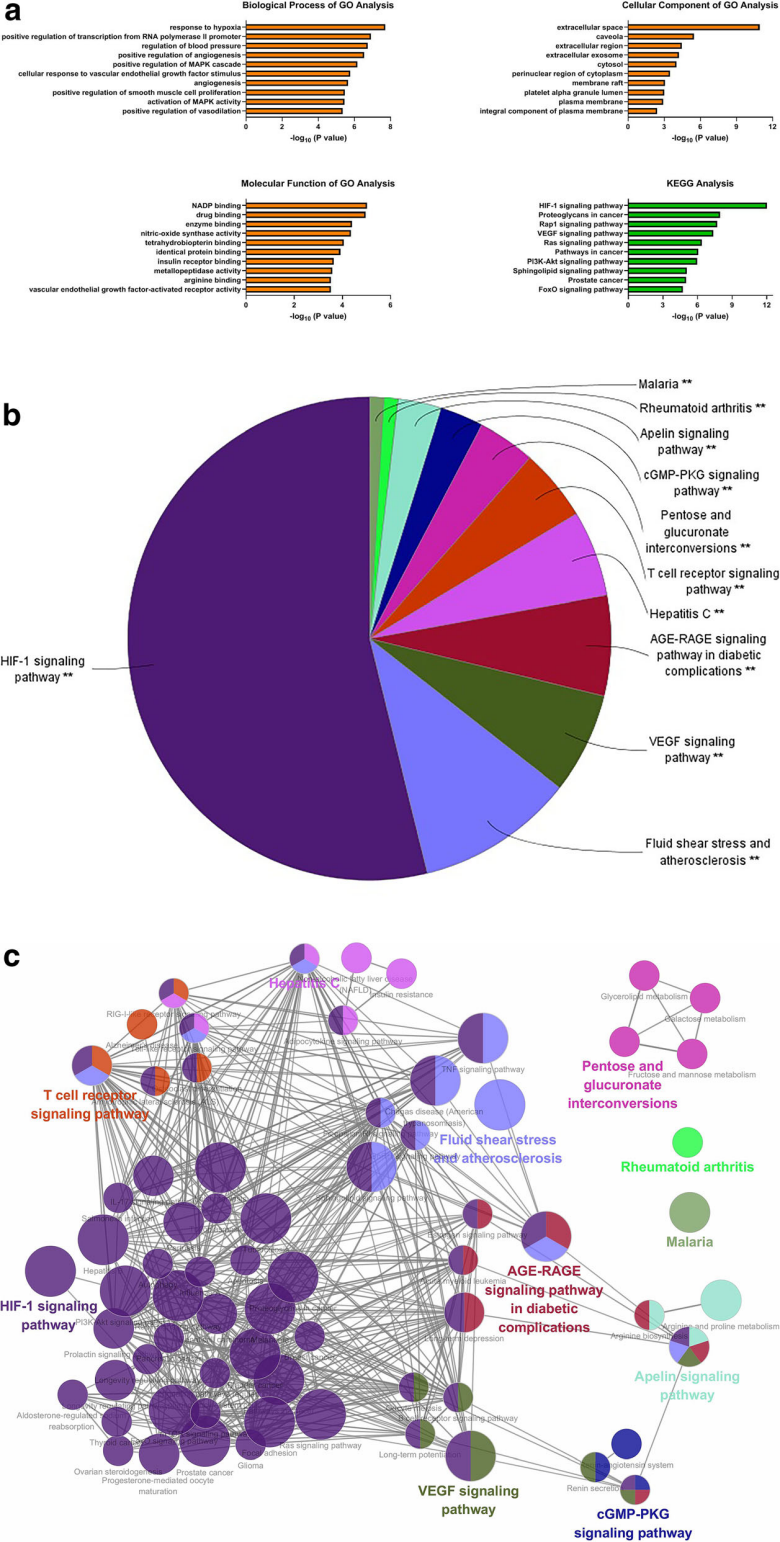
Functional enrichment of GO terms and KEGG pathways from DAVID and ClueGo. **a** GO terms and KEGG pathways from DAVID. **b** The pie chart from ClueGo. It shows the enriched signaling pathway categories based on the kappa coefficient, including HIF-1 signaling pathway, fluid shear stress and atherosclerosis, VEGF signaling pathway, AGE-RAGE signaling pathway in diabetic complications, hepatitis C, T cell receptor signaling pathway, pentose and glucoronate interconversions, cGMP-PKG signaling pathway, apelin signaling pathway, rheumatoid arthritis, and malaria. **c** The functional enrichment network from ClueGo. The node represents the signaling pathway, and the size of each node represents the enrichment significance of each signaling pathway. The larger the node is, the more significant the pathway is. The line represents the correlation between functions, and the thickness of each line represents the kappa coefficient between functions. The thicker the line is, the greater the kappa coefficient is

Subsequently, the 67 candidate targets were imported into DAVID database to perform the functional enrichment analysis. The significant changed GO terms and KEGG pathways were evaluated based on the *P* value (Fig. 4a). The top three GO terms of biological process were response to hypoxia, positive regulation of transcription from RNA polymerase II promoter, and regulation of blood pressure. The top three GO terms of cellular component were extracellular space, caveola, and extracellular region. The top three GO terms of molecular function were NADP binding, drug binding, and enzyme binding. The top three KEGG pathways were HIF-1 signaling pathway, proteoglycans in cancer, and rap1 signaling pathway. From the analysis results of biological process and signaling pathway, we speculated that hypoxia response and hypoxia-related HIF-1 signaling pathway might be the important molecular mechanisms of THSWD against DR.

On the other hand, the 67 candidate targets were further imported into ClueGo plug-in to perform KEGG pathway analysis. As shown in Fig. 4b and c, signaling pathways were divided into 11 enriched categories based on the kappa coefficient, including HIF-1 signaling pathway, fluid shear stress and atherosclerosis, VEGF signaling pathway, AGE-RAGE signaling pathway in diabetic complications, and so on. We found that HIF-1 signaling pathway was the most significant enriched category. It further indicated that HIF-1 signaling pathway played a vital role in the treatment of THSWD against DR.

#### Core signaling pathway network of THSWD against DR

**Fig. 5:**
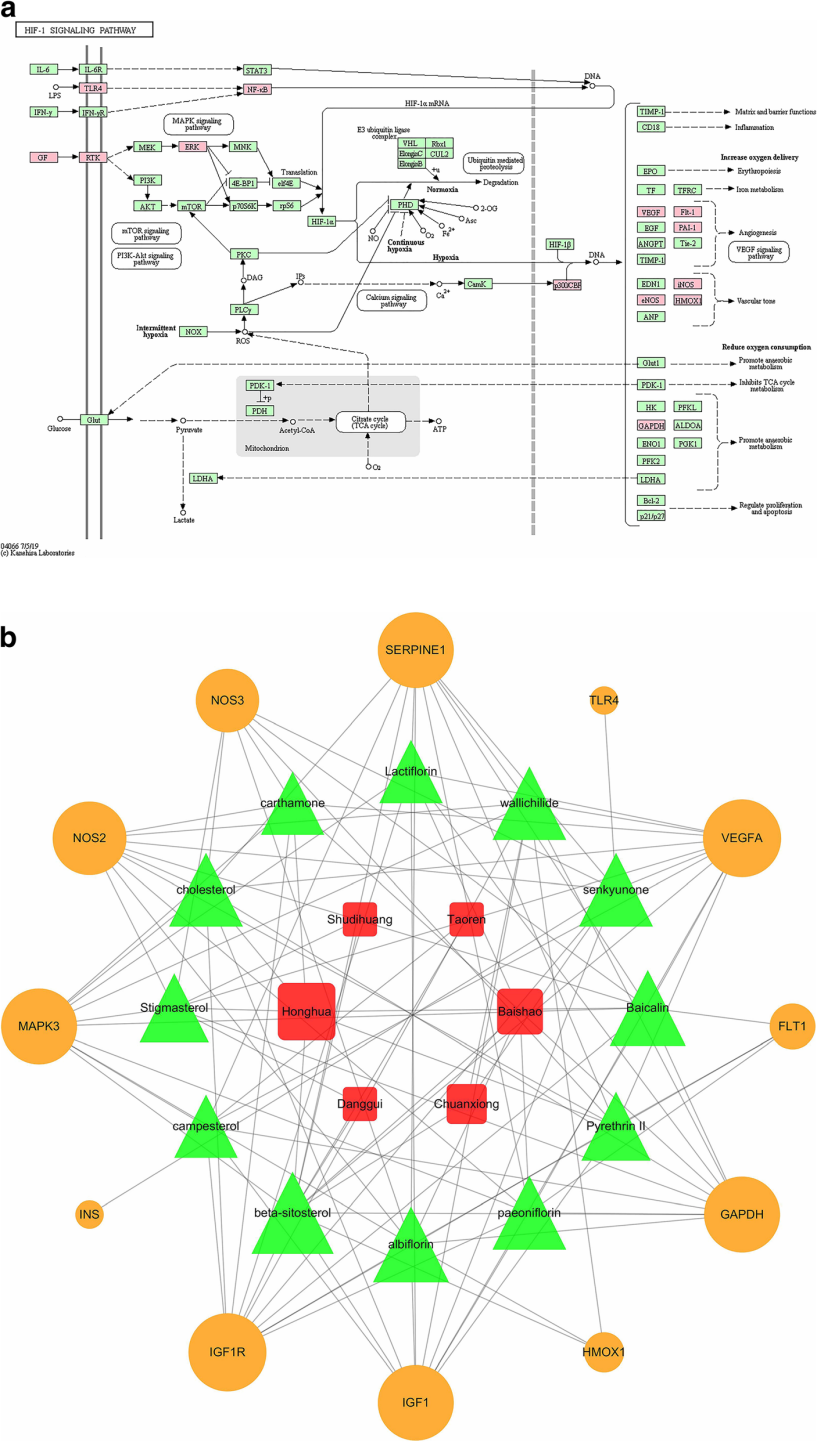
The core HIF-1 signaling pathway in THSWD against DR. **a** The KEGG pathway of HIF-1 signaling pathway. The targets of THSWD against DR were labelled as red. **b** The “herb-ingredient-target” network of HIF-1 signaling pathway. The red square represents herbs in THSWD, the green triangle represents active ingredients of those herbs, and the orange circle represents targets of THSWD against DR. The size of each node represents the correlation degree with other nodes, and the larger the node is, the stronger the correlation is

Considering the importance of HIF-1 signaling pathway in the treatment of THSWD, we further analyzed the targets and active ingredients of THSWD against DR in HIF-1 signaling pathway. There were 14 targets found in HIF-1 signaling pathway, including EP300, FLT1, GAPDH, HMOX1, IGF1, IGF1R, INS, MAPK3, NFKB1, NOS2, NOS3, SERPINE1, TLR4 and VEGFA, which were labelled as red in Fig. 5a. Notably, except for HMOX1 and TLR4, the other 12 targets all belonged to the core target group obtained from PPI network above (Fig. 3), which further proved the important role of HIF-1 signaling pathway. In addition, as shown in Fig. 5a, more targets of THSWD against DR focused on the effect of angiogenesis and vascular tone, which were closely related to DR.

Subsequently, the “herb-ingredient-target” network of HIF-1 signaling pathway was analyzed by Cytoscape software. According to the screening criteria of the degree score above the average value, the core network was filtered out (Fig. 5b). The core targets in HIF-1 signaling pathway were IGF1R, VEGFA, MAPK3, IGF1, SERPINE1, GAPDH, NOS2, NOS3, FLT1, HMOX1, TLR4 and INS. The core active ingredients were beta-sitosterol, baicalin, albiflorin, cholesterol, wallichilide, senkyunone, paeoinflorin, stigmasterol, pyrethrin II, carthamone, lactiflorin and campesterol. THSWD might alleviate the symptoms of DR via those core active ingredients and targets.

### The influence of THSWD on key molecules of HIF-1 signaling pathway

#### Quality control of THSWD

**Fig. 6:**
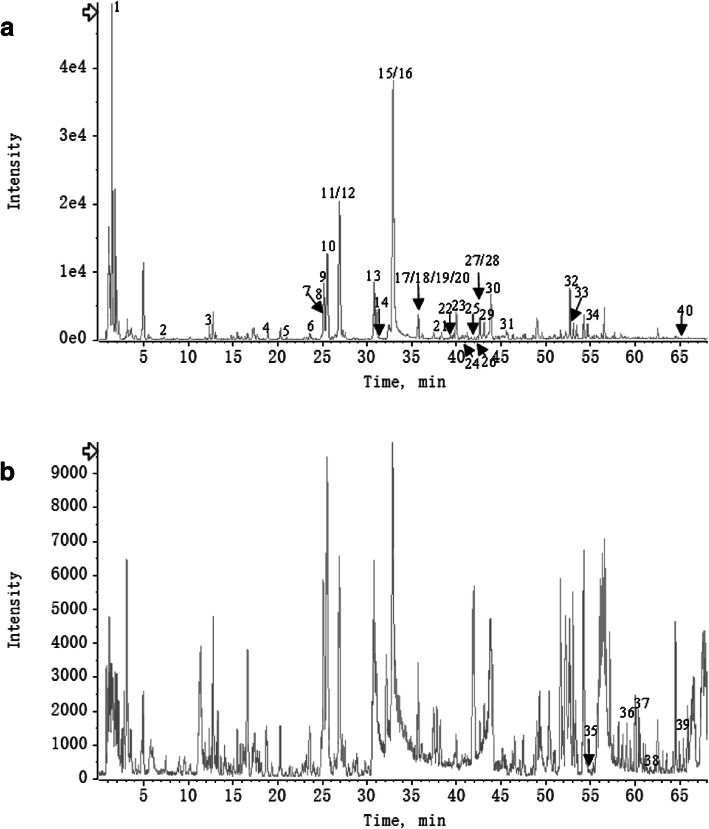
LC/MS chromatogram of THSWD in negative and positive mode (**a** and **b**)

THSWD was analyzed by LC/MS in negative and positive-ion mode (Fig. 6a and b). Based on the database of Natural Products HR-MS/MS Spectral Library and the relevant references, 40 compounds were identified in THSWD (Supplementary Table 4). These results provided the scientific basis for other researchers to explore the ingredients and mechanisms of THSWD in further studies.

#### Changes of expression levels of key molecules in THSWD against DR

**Fig. 7:**
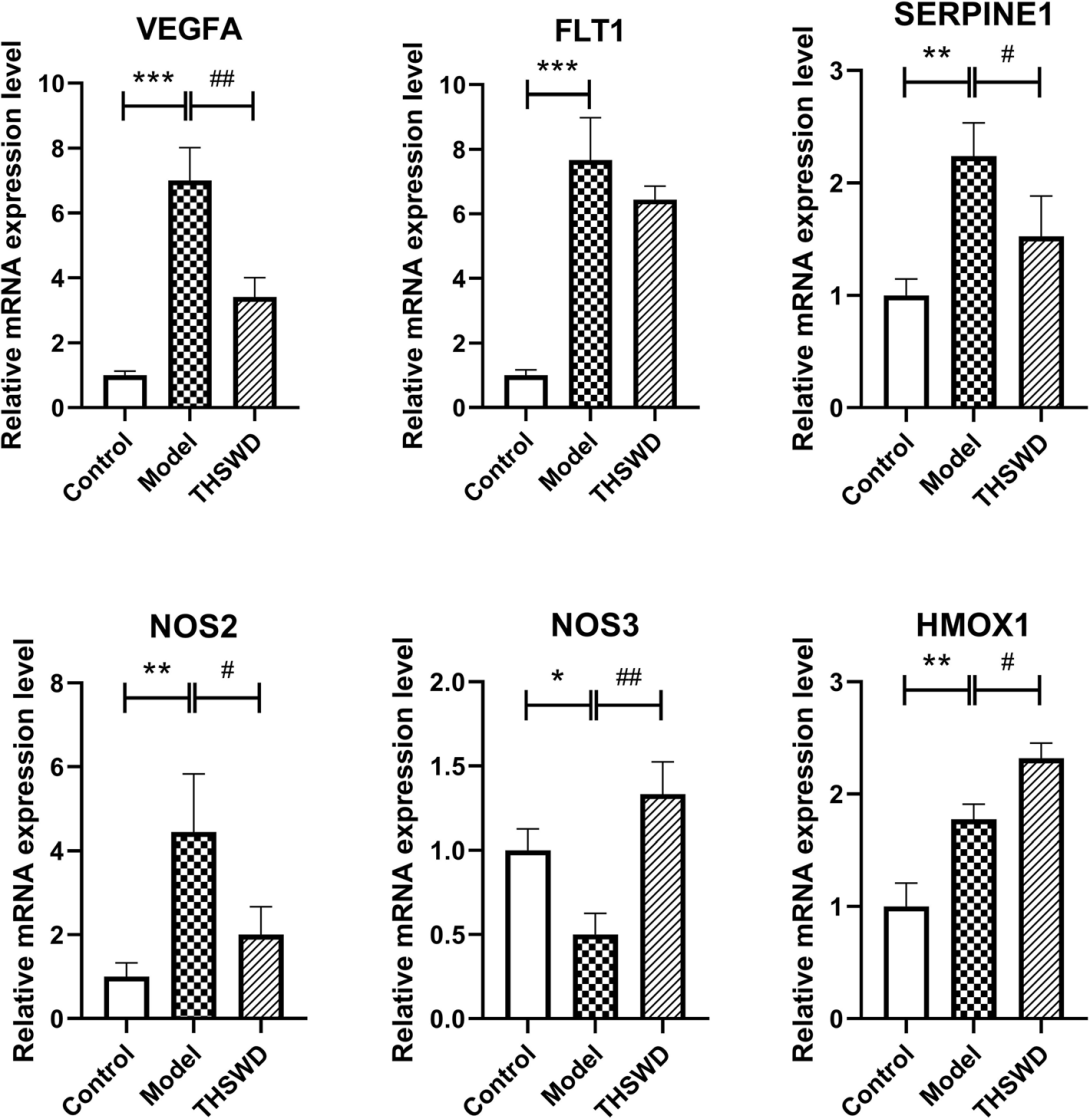
Relative mRNA expression levels of key genes in THSWD against DR. All data were measured by qRT-PCR. *GAPDH* was used as the internal control. *N* = 3 in each group. Values were expressed as mean ± standard deviation (SD). Significant differences were analyzed by One-Way ANOVA with Dunnett post hoc test. * *P* < 0.05, ** *P* < 0.01, *** *P* < 0.001 (vs. control group); # *P* < 0.05, ## *P* < 0.01, ### P < 0.001 (vs. model group)

Compared with control group, the mRNA expression levels of *VEGFA*, *FLT1*, *SERPINE1*, *NOS2* and *HOMX1* were significantly up-regulated in model group (*P* < 0.01), and *NOS3* was significantly down-regulated (*P* < 0.05). Compared with model group, the mRNA expression levels of *VEGFA*, *SERPINE1* and *NOS2* were significantly down-regulated in THSWD group (*P* < 0.05), and *NOS3* and *HMOX1* were significantly up-regulated (*P* < 0.05). (Fig. 7).

## Discussion

DR is caused by microangiopathy and capillary closure, which results in the breakdown of the blood-retinal barrier with retinal hemorrhage, exudate and edema formation, and macular edema [24]. Several studies have reported that THSWD had a good clinical effect on the treatment of DR. [13] However, fewer researches could explain the mechanism of THSWD against DR at the molecular level. In our study, according to network analysis and experimental validation, we found that HIF-1 signaling pathway and some related targets were mainly involved in the treatment of THSWD against DR.

The normal microvessel wall is based on the basement membrane. Hyperglycemia induces injury to vascular endothelial cells, which leads to progressive thickening of the basement membrane and obstruction of the involved microvascular, thus resulting in tissue hypoxia and increased lesions [25]. Retinal hypoxia is a common factor in the development of DR. [26] Hypoxia inducible factor-1 (HIF-1) is dysregulated following hypoxia [27]. HIF-1 is the transcription factor that improves hypoxia adaptation and plays an important functional role in a wide range of ischemic and inflammatory diseases [28]. Previous studies have reported that suppressing HIF-1 signaling pathway could contribute to the remission of DR. [29, 30] From our results, the pharmaceutical mechanism of THSWD against DR might be associated with the suppression of HIF-1 signaling pathway.

Vascular endothelial growth factor A (VEGFA) was the core targets of THSWD against angiogenesis of DR via HIF-1 signaling pathway from our results. Hypoxia is the most important trigger for VEGF upregulation mainly through HIF-1 [31]. VEGF is a highly potent proangiogenic agent which induces retinal angiogenesis, the major pathological characteristics of DR. [32] Previous studies have reported that some core active ingredients we found in HIF-1 pathway (Fig. 6b) could regulate the expression of VEGF. For example, some researchers proved that baicalin [33, 34], paeoniflorin [35–38] and stigmasterol [39] reduced the expression of VEGF and had a powerful effect on anti-angiogenesis, which were consistent with our findings.

Serpin family E member 1 (SERPINE1; plasminogen activator inhibitor 1, PAI1) was another core targets of THSWD against angiogenesis. SERPINE1, the inhibitor of the urokinase-type plasminogen activator (uPA), participates in the processes of VEGF-initiated angiogenesis by promoting the migration of endothelial cells [40]. It has been reported that SERPINE1 expression was upregulated in the retina of mice with retinopathy and angiogenesis significantly alleviated in SERPINE1-knockdown mice [41]. Baibalin [42] and paeoniflorin [43] have been proved to inhibit the production of SERPINE1, but the researches mainly focused on antithrombotic and antifibrotic activities.

Nitric oxide synthase 2 (NOS2; inducible NOS, iNOS) and nitric oxide synthase 3 (NOS3; endothelial NOS, eNOS) were also the core targets of THSWD against DR in HIF-1 signaling pathway. NOS are enzymes that catalyze the conversion of L-arginine to L-citrulline and nitric oxide (NO) [44], a small free radical with critical signaling roles in physiology and pathophysiology [45]. In different circumstances, NOS enzyme expression and function are highly specific to each isoform. NOS2/iNOS is not expressed under normal conditions, while in human immune responses, NOS2 expression will be induced by proinflammatory cytokines. NOS3/eNOS is expressed almost exclusively in the endothelium under basal conditions, while in hypoxia, the expression will reduce, which further influences vascular tone, hemostasis and angiogenesis [46]. A long-standing challenge for researchers in the NOS field has been to provide plausible mechanism to explain opposite biological responses to NO under seemingly similar circumstances resulting from the complex mechanisms of NO. In fact, differences in tissue O_2_ concentrations will partially determine the expressions of NOS enzymes and the steady-state concentration of NO, subsequently dictating what cellular targets interacted with. The types and distributions of cellular targets will determine cell phenotype, ultimately leading to positive or negative effects on disease outcome [47]. In our study, the expression of NOS2 mRNA was significantly down-regulated in THSWD group compared to model group, while the expression of NOS3 mRNA was significantly up-regulated in THSWD group. However, the concentration of NO and further biological responses to NO did not be detected. Thus, the effects and mechanisms of THSWD on NOS enzymes need to be further explored.

In addition, heme oxygenase 1 (HMOX1) was also the targets of THSWD against DR in HIF-1 signaling pathway. HMOX1 is an intracellular enzyme that catalyzes the oxidation of heme to generate carbon monoxide (CO), ferrous iron, and biliverdin, which is subsequently converted to bilirubin. These products can display multiple actions, including anti-oxidation, anti-inflammation and anti-apoptosis [48]. Under basal conditions, HMOX1 is expressed at low levels in most tissues, while in response to various pathophysiological stresses/stimuli, the expression will be highly induced. Thus, HMOX1 induction is thought to be an adaptive defense mechanism in order to protect the human body against injury in many disease situations [49]. From our results, HMOX1 was found significantly up-regulated by THSWD. Previous study reported that beta-sitosterol, one core active ingredient of THSWD, could enhance the expression of HMOX1 [50]. It suggested that THSWD and its ingredients might play a protective role in DR via regulating the expression of HMOX1.

## Conclusions

Taken together, this study uncovered the multi-ingredient and multi-target mechanisms of THSWD against DR by combining network analysis and experimental validation. It was found that THSWD was capable of regulating HIF-1 signaling pathway and other important pathways, and had powerful effects of anti-angiogenesis, anti-oxidation and so on. This study explained the complex ingredients and pharmacological mechanisms of THSWD, and identified potential therapeutic targets and signaling pathways, which could provide a theoretical basis for the application of THSWD and the development of new drugs for the treatment of DR.

## Supplementary information

**Additional file 1: Table S1.** Sixty one active ingredients found in Taohong Siwu decoction (THSWD) after overlapped ingredients were subtracted. The active ingredients were obtained from Traditional Chinese Medicine Systems Pharmacology (TCMSP) database (http://lsp.nwu.edu.cn/tcmsp.php) according to meeting the criteria of oral bioavailability (OB ≥ 30%) and drug-likeness (DL ≥ 0.18). **Table S2.** Two thousand three hundred forty targets of active ingredients of THSWD. The targets were identified using ChemMapper database (http://lilab.ecust.edu.cn/chemmapper/) according to the criteria of 3D structure similarity above 0.85 and prediction score above 0 and PharmMapper (http://lilab.ecust.edu.cn/pharmmapper/index.php) databases according to the target pharmacophore approach. **Table S3.** Two hundred sixty three diabetic retinopathy (DR)-associated genes. DR-associated genes were searched from DisGeNET database (http://www.disgenet.org/web/DisGeNET/menu/home), DrugBank database (https://www.drugbank.ca/), and Therapeutic Target Database (TTD) (https://db.idrblab.org/ttd/). **Table S4.** Characterization of chemical constituents of THSWD by UPLC–ESI-Q-TOF/MS.

## Data Availability

The datasets used and/or analyzed during the current study are available in the Supplementary materials.

## Abbreviations

BS: Baishao, Paeoniae Radix Alba
CO: Carbon monoxide
CX: Chuangxiong, Chuanxiong Rhizoma
DG: Ganggui, Angelicae Sinesis Radix
DR: Diabetic retinopathy
GO: Gene ontology
HH: Honghua, Carthami Flos
HIF-1: Hypoxia inducible factor-1
HMOX1: Heme oxygenase 1
hRMECs: Human retinal microvascular endothelial cells
KEGG: Kyoto Encyclopedia of Genes and Genomes
LC/MS: Liquid chromatography/mass spectrometry
NO: Nitric oxide
NOS2: nitric oxide synthase 2
NOS3: nitric oxide synthase 3
PPI: Protein-protein interaction
qRT-PCR: Quantitative real-time PCR
SD: Standard deviation
SDH: Shudihuang, Rehmanniae Radix Praeparata
SERPINE1: serpin family E member 1
TCM: Traditional Chinese medicine
TCMSP: Traditional Chinese Medicine Systems Pharmacology
THSWD: Taohong Siwu decoction
TR: Taoren, Persicae Semen
TTD: Therapeutic Target Database
uPA: Urokinase-type plasminogen activator
VEGF: Vascular endothelial growth factor
