# Supplementary material for: Uncovering the protective mechanism of Taohong Siwu decoction against diabetic retinopathy via HIF-1 signaling pathway based on network analysis and experimental validation
Source: BMC Complement Med Ther. 2020 Oct 6;20:298. doi: 10.1186/s12906-020-03086-0 (PMC7542117; doi:10.1186/s12906-020-03086-0)
Supplement: Supplementary file 1 — Additional file 1: Table S1. Sixty one active ingredients found in Taohong Siwu decoction (THSWD) after overlapped ingredients were subtracted. The active ingredients were obtained from Traditional Chinese Medicine Systems Pharmacology (TCMSP) database (http://lsp.nwu.edu.cn/tcmsp.php) according to meeting the criteria of oral bioavailability (OB ≥ 30%) and drug-likeness (DL ≥ 0.18). Table S2. Two thousand three hundred forty targets of active ingredients of THSWD. The targets were identified using ChemMapper database (http://lilab.ecust.edu.cn/chemmapper/) according to the criteria of 3D structure similarity above 0.85 and prediction score above 0 and PharmMapper (http://lilab.ecust.edu.cn/pharmmapper/index.php) databases according to the target pharmacophore approach. Table S3. Two hundred sixty three diabetic retinopathy (DR)-associated genes. DR-associated genes were searched from DisGeNET database (http://www.disgenet.org/web/DisGeNET/menu/home), DrugBank database (https://www.drugbank.ca/), and Therapeutic Target Database (TTD) (https://db.idrblab.org/ttd/). Table S4. Characterization of chemical constituents of THSWD by UPLC–ESI-Q-TOF/MS. [file 12906_2020_3086_MOESM1_ESM.zip › Supplementary Table 4R5.docx]

**Supplementary Table 4. Characterization of chemical constituents of THSWD by UPLC–ESI-Q-TOF/MS**

| **NO** | **RT**  **（min）** | **Adduct**  **ions** | **Measured**  ***m/z*** | **Respected**  ***m/z*** | **Mass error**  **（ppm）** | **Formula** | **M.W.** | **Identification** | **MS/MS data** |
| --- | --- | --- | --- | --- | --- | --- | --- | --- | --- |
| 1 | 1.45 | [M-H]^-^ | 341.1092 | 341.1089 | 0.8 | C_12_H_22_O_11_ | 342.12 | Sucrose | 341.1042;179.0542;119.0337;89.0229 |
| 2 | 7.26 | [M-H]^-^ | 169.0152 | 169.0142 | 5.6 | C_7_H_6_O_5_ | 170.02 | Gallic acid | 169.0155;125.0246;97.0303;79.0188 |
| 3 | 12.35 | [M-H]^-^ | 282.0842 | 282.0844 | -0.7 | C_10_H_13_N_5_O_5_ | 283.09 | Guanosine | 150.0405;133.0136;108.0209 |
| 4 | 18.91 | [M-H]^-^ | 353.0861 | 353.0878 | -4.8 | C_16_H_18_O_9_ | 354.10 | Neochlorogenic acid | 191.0575;179.0356;135.0484 |
| 5 | 21.00 | [M-H]^-^ | 375.1290 | 375.1297 | -1.8 | C_16_H_24_O_10_ | 376.14 | 8-epiloganic acid | 375.1216;213.0776;169.0867;151.0765 |
| 6 | 23.59 | [M+FA-H]^-^ | 477.1612 | 477.1614 | -0.3 | C_19_H_28_O_11_ | 432.16 | Benzyl gentiobioside | 431.1517;269.0981;161.0504;101.0221 |
| 7 | 25.02 | [M-H]^-^ | 289.0713 | 289.0718 | -1.6 | C_15_H_14_O_6_ | 290.08 | Catechin | 289.0724;245.0838;109.0310 |
| 8 | 25.13 | [M-H]^-^ | 353.0883 | 353.0878 | 1.4 | C_16_H_18_O_9_ | 354.10 | Chlorogenic acid | 191.0542 |
| 9 | 25.18 | [M+FA-H]^-^ | 502.1561 | 502.1566 | -1 | C_20_H_27_NO_11_ | 457.16 | L-Amygdalin | 456.1508;323.0958;263.0758;221.0649;119.0332 |
| 10 | 25.57 | [M+FA-H]^-^ | 502.1587 | 502.1566 | 4.2 | C_20_H_27_NO_11_ | 457.16 | D-Amygdalin | 502.1535;456.1465;323.0951;221.0644 |
| 11 | 26.92 | [M-H]^-^ | 611.1630 | 611.1618 | 2 | C_27_H_32_O_16_ | 612.17 | Hydroxysafflor yellow A | 611.1598;491.1180;403.1022;325.0703;283.0585 |
| 12 | 26.93 | [M-H]^-^ | 353.0888 | 353.0878 | 2.8 | C_16_H_18_O_9_ | 354.10 | Cryptochlorogenic acid | 191.0552;179.0324;173.0440;135.0438 |
| 13 | 30.81 | [M+FA-H]^-^ | 525.1595 | 525.1614 | -3.6 | C_23_H_28_O_11_ | 480.16 | Albiflorin | 525.1570;479.1516;357.1179 |
| 14 | 31.55 | [M-H]^-^ | 289.0708 | 289.0718 | -3.3 | C_15_H_14_O_6_ | 290.08 | Epicatechin | 123.0436 |
| 15 | 32.91 | [M-H]^-^ | 495.1489 | 495.1508 | -3.8 | C_23_H_28_O_12_ | 496.16 | Oxypaeoniflora | 449.1402;327.1057;165.0547;121.0291 |
| 16 | 32.92 | [M+FA-H]^-^ | 525.1611 | 525.1614 | -0.5 | C_23_H_28_O_11_ | 480.16 | Paeoniforin | 449.1434;431.1297;327.1053 |
| 17 | 35.62 | [M-H]^-^ | 193.0509 | 193.0506 | 1.4 | C_10_H_10_O_4_ | 194.06 | Ferulic Acid | 178.0255;158.8444;149.0602;134.0356 |
| 18 | 35.65 | [M-H]^-^ | 625.1437 | 625.1410 | 4.3 | C_27_H_30_O_17_ | 626.52 | Quercetin 3-O-sophoroside | 625.1395;463.0846;301.0290 |
| 19 | 35.68 | [M-H]^-^ | 785.2517 | 785.2510 | 0.9 | C_35_H_46_O_20_ | 786.26 | Purpureaside C | 785.2530;623.2267L;161.0244 |
| 20 | 35.75 | [M-H]^-^ | 771.2015 | 771.1989 | 3.3 | C_33_H_40_O_21_ | 772.21 | 6-Hydroxykaempferol-3-O-β-rutinoside-6-O-β-D-glucoside | 771.1969;609.1423;463.0918;301.0274 |
| 21 | 38.20 | [M-H]^-^ | 799.2651 | 799.2666 | -1.9 | C_36_H_48_O_20_ | 800.27 | Jionoside A1 | 799.2627;623.2181;175.0376 |
| 22 | 39.40 | [M-H]^-^ | 463.0880 | 463.0882 | -0.4 | C_21_H_20_O_12_ | 464.10 | Myricitrin | 463.0791;316.0209;271.0182 |
| 23 | 39.99 | [M-H]^-^ | 631.1650 | 631.1668 | -2.9 | C_30_H_32_O_15_ | 632.17 | Galloyl paeoniflorin | 631.1679;613.1636;465.1405;399.0937 |
| 24 | 40.46 | [M-H]^-^ | 463.0875 | 463.0882 | -1.5 | C_21_H_20_O_12_ | 464.10 | Quercetin-3-O-β-D-glucoside | 463.0930;300.0283;271.0229 |
| 25 | 42.01 | [M-H]^-^ | 813.2784 | 813.2823 | -4.8 | C_37_H_50_O_20_ | 814.29 | Jionoside B1 | 813.2792;175.0385 |
| 26 | 42.25 | [M-H]^-^ | 515.1244 | 515.1195 | 9.5 | C_25_H_24_O_12_ | 516.46 | Isochlorogenic acid B | 299.0536;173.0418 |
| 27 | 42.65 | [M-H]^-^ | 593.1497 | 593.1512 | -2.5 | C_27_H_30_O_15_ | 594.16 | Kaempferol-3-O-rutinoside | 593.1524;285.0422;255.0338 |
| 28 | 42.69 | [M-H]^-^ | 515.1194 | 515.1195 | -0.2 | C_25_H_24_O_13_ | 516.46 | Isochlorogenic acid A | 515.1233;353.0851;179.0325;135.0438 |
| 29 | 43.12 | [M-H]^-^ | 623.1615 | 623.1618 | -0.4 | C_28_H_32_O_16_ | 624.17 | Isorhamnetin-3-O-neohespeidoside | 623.1627;315.0483;300.0264;272.0396 |
| 30 | 43.88 | [M+FA-H]^-^ | 525.1618 | 525.1614 | 0.8 | C_23_H_28_O_11_ | 480.16 | Isomer of paeoniforin | 525.1671;479.1573;121.0291 |
| 31 | 45.59 | [M-H]^-^ | 515.1192 | 515.1195 | -0.6 | C_25_H_24_O_14_ | 516.46 | Isochlorogenic acid C | 515.1249;353.0903;173.0455;135.0449 |
| 32 | 52.71 | [M+FA-H]^-^ | 629.1905 | 629.1876 | 4.6 | C_30_H_32_O_12_ | 584.19 | Benzoylpaeoniflorin | 629.1737;583.1759;553.1643;535.1581;431.1299 |
| 33 | 53.11 | [M+FA-H]^-^ | 629.1859 | 629.1876 | -2.7 | C_30_H_32_O_12_ | 584.19 | Benzoylalbiflorin | 629.1878;583.2612;463.2067;343.1486 |
| 34 | 54.68 | [M-H]^-^ | 329.2340 | 329.2333 | 2 | C_18_H_34_O_5_ | 330.24 | Trihydroxyoctadecaenoic acid | 329.2308;229.1423;211.1325 |
| 35 | 54.87 | [M+H]^+^ | 191.1058 | 191.1067 | -4.5 | C_12_H_14_O_2_ | 190.10 | Butylphthalide | 191.1053;173.0972;135.0440 |
| 36 | 59.16 | [M+H]^+^ | 193.1210 | 193.1223 | -6.8 | C_12_H_16_O_2_ | 192.12 | Senkyunolide A | 147.1161;137.0587;91.0529;77.0375 |
| 37 | 60.81 | [M+H]^+^ | 191.1062 | 191.1067 | -2.4 | C_12_H_14_O_2_ | 190.10 | (E)-Ligustilide | 191.1071;173.0973;145.1127 |
| 38 | 61.67 | [M+H]^+^ | 191.1055 | 191.1067 | -6.1 | C_12_H_14_O_2_ | 190.10 | Ligustilide | 191.1071;173.0962;145.1008 |
| 39 | 62.54 | [M+H]^+^ | 381.2058 | 381.2060 | -0.6 | C_24_H_28_O_4_ | 380.20 | Levistilide A or isomer | 209.0565;191.1050;173.1049 |
| 40 | 65.95 | [M+H]^+^ | 381.2030 | 381.2060 | -1.8 | C_24_H_28_O_4_ | 380.20 | Levistilide A or isomer | 191.1052;183.1142;173.0968 |
